# Pyrolysis of Chilean Southern Lignocellulosic Biomasses: Isoconversional Kinetics Analysis and Pyrolytic Products Distribution

**DOI:** 10.3390/polym15122698

**Published:** 2023-06-16

**Authors:** Cristian Cerda-Barrera, Kevin J. Fernández-Andrade, Serguei Alejandro-Martín

**Affiliations:** 1 Department of Industrial Processes, Universidad Católica de Temuco, Temuco 4780000, Chile; ccerda@uct.cl; 2 Laboratory of Gas Chromatography and Analytical Pyrolysis, Universidad del Bío-Bío, Concepción 4030000, Chile; kevin.fernandez2101@alumnos.ubiobio.cl; 3 Wood Engineering Department, Engineering Faculty, Universidad del Bío-Bío, Concepción 4030000, Chile

**Keywords:** biomass, analytical pyrolysis, isoconversional methods, thermogravimetric analysis

## Abstract

Biomass provides potential benefits for obtaining value-added compounds instead of straight burning; as Chile has forestry potential that supports such benefits, it is crucial to understand the biomasses’ properties and their thermochemical behaviour. This research presents a kinetic analysis of thermogravimetry, and pyrolysis of representative species in the biomass of southern Chile, heating biomasses at 5 to 40 °C·min^−1^ rates before being subjected to thermal volatilisation. The activation energy (Ea) was calculated from conversion using model-free methods (Flynn–Wall–Ozawa (FWO), Kissinger–Akahira–Sunose (KAS), and Friedman (FR)), as well as the Kissinger method based on the maximum reaction rate. The average Ea varied between KAS 117 and 171 kJ·mol^−1^, FWO 120–170 kJ·mol^−1^, and FR 115–194 kJ·mol^−1^ for the five biomasses used. *Pinus radiata* (PR) was identified as the most suited wood for producing value-added goods based on the Ea profile for the conversion (α), along with *Eucalyptus nitens* (EN) for its high value of reaction constant (k). Each biomass demonstrated accelerated decomposition (an increase in k relative to α). The highest concentration of bio-oil containing phenolic, ketonic, and furanic compounds was produced by the forestry exploitation biomasses PR and EN, demonstrating the viability of these materials for thermoconversion processes.

## 1. Introduction

Using raw biomass as an energy source in conventional domestic stoves results in incomplete combustion, harmful pollutant emissions, and low thermal efficiency [1]. Pyrolysis (occurring at the thermochemical process early stages in an inert atmosphere) is a promising method for utilising biomass without reducing its definition to fuel but converting it into a diverse range of chemical compounds [1,2]. Although biomass pyrolysis liquid (bio-oil) can be used as fuel, it also contains high-value-added compounds (furfural, acetic acid, and phenol, among others). Thus, increasing selectivity towards those compounds requires understanding the thermal and kinetic behaviour of the biomass when used as feedstock, significantly impacting product distribution, as it depends on physicochemical properties [3]. Such properties of lignocellulosic biomass, mainly wood, vary considerably between species, planting sites, and tree parts [4], implying differences in thermal behaviour. Then, before studying the thermochemical decomposition of biomass in depth, relationships between samples and the formed compounds and their decomposition must be investigated through kinetic analysis. In wood pyrolysis research, having comprehensive data on decomposition kinetic parameters calculated by various methods is extremely useful for establishing starting points for analysis and shortening research times.

Thermogravimetric analysis (TGA) contributes to studying the thermochemical decomposition of biomass during pyrolysis, using an appropriate experimental setup, allowing convincing conclusions on reaction kinetics. TGA was used previously for palm oil residues [5], corn [6], coffee beans [7], and some types of wood such as oak [8], pine [9], spruce and birch [10], and even the main components of biomass, cellulose, hemicellulose, and lignin [9]. For the obtained activation energies (Ea), values go from 70 to 250 kJ·mol^−1^, as a consequence of the lignin content and spatial arrangement in the biomass, crystalline cellulose fractions, oxygen, moisture, and ash contents, all of which can strongly displace the TGA peaks of higher mass loss. Changes in biomass chemical composition were significant, with differences in lignin content of up to 30%, ash content of 8% [7,9], and fixed carbon of 10% [10], demonstrating the dependence of chemical composition on biomass type. Furthermore, wood species can be classified as hardwoods and softwoods based on their chemical composition and fibre spatial arrangement. This is relevant in Chile, with silvicultural exploitation (4494 million m^3^ in 2020 [11]), mainly pine and eucalyptus, albeit in the presence of native forest species (Oak and Coigüe), generating large amounts of residues (25% of annual exploitation [11]). Thus, the significant differences between species and their behaviour during conversion into value-added chemical products via thermal decomposition kinetics are quite interesting.

TGA data obtained with proper control of operating conditions and mathematical models provide relevant information on the thermal biomass decomposition kinetics, allowing activation energy calculations. Applying mathematical models to TGA data is a well-known method for calculating Ea. The Kinetics Committee of the International Confederation of Thermal Analysis and Calorimetry (ICTAC) recommends performing tests in a non-isothermal state at different heating rates [12], which complicates the application of model-free approaches. Then, researchers use so-called isoconversion methods because they do not require prior assumptions about the reaction mechanism to calculate the kinetic parameters, reducing the possibility of errors in the reaction model selection [12]. Kissinger–Akahira–Sunose (KAS) [13,14], Flynn–Wall–Ozawa (FWO) [15,16], Friedman (FR) [17], and Starink (ST) [18] are among the most used methods, and their accuracy varies depending on the researchers’ criteria. Moreover, as these methods are based on calculating Ea and ignore the dependence on the pre-exponential factor (A_0_), ICTAC recommends obtaining the compensation factor and considering the Master Plot to ensure accurate conclusions [19,20], allowing an accurate approximation of the kinetic triplet determination for the different types of biomass or study conditions.

Despite extensive research into biomass thermochemical decomposition, there are still some gaps in the relationship between the generated chemical compounds and the required energy for decomposition reactions in different wood species. Considering the former scenario, this article reports a study of pyrolysis (fast and slow) of local Chilean biomass species, combined with thermogravimetric measurements and kinetic parameters calculation via isoconversional methods (KAS, FWO, and FR) in comparison to the Kissinger model (the maximum temperature at maximum reaction rate). Such comparisons validated the data consistency for identifying the kinetic triplet among all the kinetic parameters obtained, correlating product distribution in laboratory and analytical-scale pyrolysis.

## 2. Materials and Methods

### 2.1. Samples Preparation

Five species of lignocellulosic biomass frequently used as solid fuel in southern Chile were investigated. Four samples of hardwoods: Oak (*Nothofagus obliqua,* NO), Coigüe (*Nothofagus domberyi,* ND), Eucalyptus (*Eucalyptus nitens*, EN), and Ulmo (*Eucryphia cordifolia*, EC); and Pine (*Pinus radiata*, PR) as softwood, were collected from the Araucanía Cordilleran Area (IX Region, Chile). The samples were debarked, milled, sieved ($d_{p}=250\;µm$), oven-dried at 105 °C, and finally stored in a desiccator for further use. 

### 2.2. Proximate Analysis

Proximate analysis of biomass samples (5~6 mg) was performed according to the ASTM E1131 standard [21], allowing the determination of moisture, volatile material, fixed carbon, and ash content in a single experiment. The samples were subjected to continuous heating, at a constant flow of 40 mL·min^−1^, in three stages: (1) drying at an inert atmosphere of N_2_ at 10 °C·min^−1^ up to 105 °C and kept for 30 min, (2) inert atmosphere (N_2_, 20 °C·min^−1^) up to 800 °C and kept it for 5 min to eliminate all volatile material, (3) change to the reactive atmosphere (Air, 20 °C·min^−1^) up to 1000 °C to oxidate the fixed carbon and finally obtain ash content from the residual mass. 

### 2.3. Gross Calorific Value (GCV)

The gross calorific values (constant volume) were determined according to EN/TS 14918:2005(E) solid biofuels method, using a Parr 6200 Isoperibol Calorimeter. Samples were one gram of biomass: as received, dried for 24 at 105 °C and biochar obtained from pyrolysis at 350 °C and 30 min.

### 2.4. Chemical Analysis

The biomass samples were analysed using the standard TAPPI methods to determine the content of extractives, lignin, and polysaccharides (cellulose and holocellulose). First, the extractives content was determined using the TAPPI T-212 om-93 standard, using 10 g of biomass (<0.250 mm) with an ethanol–benzene solution in a Soxhlet extractor. Then, after liquid phase remotion, the solid residue was filtered, washed with ethanol, and finally with hot water until the remotion of solvent traces. The mass loss of treated biomass contributes to the calculations of extractive contents. The lignin content was determined by the TAPPI standard T 222 om-98 method. Thus, the extractive-free samples were treated with sulphuric acid (72% vol.) to hydrolyse the holocelluloses, isolating the lignin. Next, the obtained residue was filtered and washed with hot water until all residual acid was undetected. Finally, the sample was dried at 105 °C to obtain acidic insoluble lignin content (Klason lignin). The holocellulose content was determined using NaClO2, acetic acid, and water mixtures, corresponding to the sum of cellulose and hemicelluloses.

### 2.5. Thermogravimetric Analysis (TGA)

Thermogravimetric tests of biomass samples were conducted in a thermobalance (TG-60H, Shimadzu Japan), using nitrogen as purge gas (purity = 99.999%; 40 mL·min^−1^). An inert atmosphere avoids oxygen presence, removes condensable products from pyrolysis, and minimises secondary interactions with generated residue at high temperatures. The tests were carried out in a dynamic regime (non-isothermal) using four heating rates (5, 10, 20, and 40 °C·min^−1^), starting from 105 °C up to the final temperature of 900 °C, using previously dried samples at 105 °C for 10 min (10 °C·min^−1^ from room temperature, 40 mL·min^−1^). Around 5–6 mg of biomasses sample was placed into an α-alumina crucible, using a second empty crucible as reference. The Indium standard (melting point at 156.6 °C) contributes to equipment calibration.

### 2.6. Processing Thermogravimetric Data

**Figure 1:**
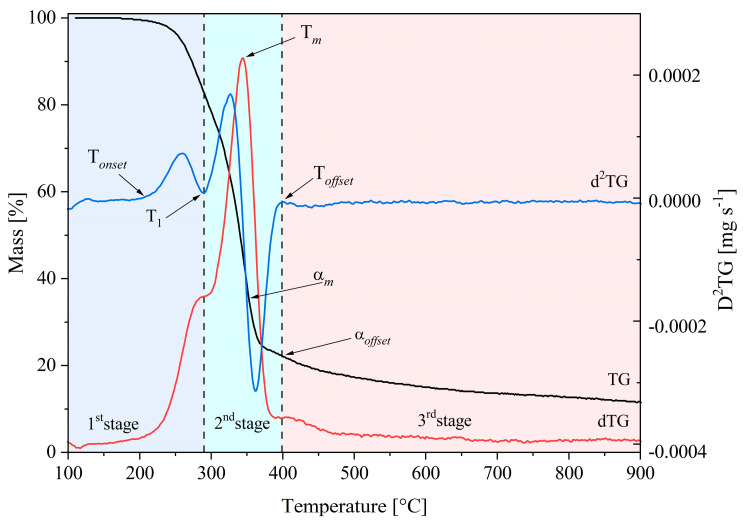
TGA parameters for biomass pyrolysis.

The Savitzky–Golay method smooths the TG and DTG curves obtained from TGA assays [22]. The Origin software (Microcal Software Inc., v6.0, Sammamish, DC, USA) calculates the kinetic parameters. TGA assays were conducted in triplicate to ensure the reproducibility of registered mass loss curves under the same experimental conditions for each biomass species and heating rate. An overlapping of mass loss curves for different assays at the same experimental conditions confirmed the reproducibility. Knowing temperature intervals of biopolymers decomposition during thermogravimetric analysis is essential to understanding pyrolysis phenomena. The second derivative of mass loss (−d^2^m/dt^2^), also known as D^2^TG, provided essential information on this topic. D^2^TG registered values close to zero established the onset (T*_onset_*) and term (T*_offset_*) temperatures of the involved stages, as Figure 1 shows. 

The T*_onset_* value indicates the starting temperature of the material volatilisation process, and T*_offset_* indicates the temperature to complete the active pyrolysis stage. At higher temperatures than T*_offset_*, a much slower weight loss stage (passive pyrolysis) begins. The conversion value (α*_offset_*) at this temperature quantifies the released volatile material in active pyrolysis. Thus, its complementary value (1 − α*_offset_*) defines the released volatiles during passive pyrolysis.

### 2.7. Kinetic Analysis

Thermal decomposition reactions can be understood through kinetic studies at various temperatures and by applying various models that describe them. Dynamic experiments using isoconversional methods provide the kinetic parameters of the studied biomass samples [12]. The global one-stage pyrolysis model assumes that the volatilisation process happens simultaneously (Equation (1)), where A represents the parent material, and B and C are the products obtained from thermal degradation. Equation (2) assumes an irreversible reaction, considering that by-products are rapidly removed from the thermobalance (via the carrier gas flow). The reaction rate constant (*k*) is affected by absolute temperature (T, K) and activation energy ($E_{a},\mathrm{kJ}\cdot \min^{-1}$), according to the Arrhenius equation (Equation (2)). The pre-exponential factor ($A_{0},{\;\min}^{-1}$) and the universal gas constant ($R,\;8.314\;\mathrm{kJ}\cdot {\mathrm{mol}}^{-1}K^{-1}$).

(1) $$A_{\left(solid\right)}\;\overset{k}{\to }\;B_{\left(solid\right)}+C_{\left(gas\right)}$$

(2) $$k=A_{0}\cdot e^{-E_{a}/RT}$$

The transformation rate of the solid to volatile products is described by Equation (3). Where the terms α, t, k(T), and f(α) represent the conversion degree of process, time, the reaction rate constant, and the reaction model, respectively. Combining Equations (2) and (3) leads to the fundamental expression of the analytical methods used to calculate the kinetic parameters based on the registered TGA results (Equation (4)). Thus, Equation (5) describes biomass degradation considering the conversion degree (α).

(3) dα/dt=k(T)·f(α)

(4) $$d\alpha /dt=A_{0}\cdot e^{-E_{a}/RT}\cdot f\left(\alpha \right)$$

(5) $$\alpha =\left(W_{0}-W\right)/\left(W_{0}-W_{f}\right)$$

The expression f(α) and their derivative are correlated to a reaction of order n with the mathematical function ${\left(1-\alpha \right)}^{n}$. Replacing f(α) and considering a linear heating rate (β=dT/dt) for non-isothermal TGA experiments, Equation (4) turns into:

(6) $$d\alpha /dT=\left(A_{0}/\beta \right)\cdot e^{-E_{a}/RT}\cdot {\left(1-\alpha \right)}^{n}$$

The Model-free methods contribute to activation energy calculations concerning progressive conversion when the reaction kinetics are assumed to be temperature-dependent, and the conversion of the starting materials to the final product only takes place in a single step. Thus, the integral form of Equation (6) is as follows:

(7) $$g\left(x\right)=\int_{0}^{\alpha }{\left[f\left(x\right)\right]}^{-1}\;d\alpha =\left(A_{o}/\beta \right)\cdot \int_{0}^{T}e^{-E_{a}/RT}\;dT$$

As the term for integral temperature has no analytical solutions, it is necessary to use the so-called isoconversional methods to approximate the kinetic parameters [18].

#### 2.7.1. Kissinger Method

The method described by Kissinger [13] for the pyrolysis reaction considers that the derivative of Equation (4) is equal to zero at the maximum reaction rate with an order equal to 1. The $\alpha_{m}$ is the conversion at $T_{m}$, and $T_{m}$ is the temperature at which mass loss occurs at the fastest pace possible. The slope of the graph (ln ($\beta /T_{m}^{2}$) vs. $1/T_{m}$) using TGA data can be used to determine the activation energy.

(8) $$\ln\left(\beta /T_{m}^{2}\right)=\ln\left(A_{0}R\cdot f^{\prime }\left(\alpha_{m}\right)/E_{a}\right)-E_{a}/RT_{m}$$

(9) $$f^{\prime }\left(\alpha_{m}\right)=n{\left(1-\alpha_{m}\right)}^{n-1}$$

#### 2.7.2. Kissinger–Akahira–Sunose (KAS) Method

Like the previous method, the KAS method [13,14] used an approximation $\left\{e^{-y^{2}/y^{2}}\;where\;y=E_{a}/RT\right\}$, previously reported by Murray and White [23], to obtain the integral temperature from Equation (7).

(10) $$\ln\left(\beta_{i}/T_{\alpha ,i}^{2}\right)=\ln\left(A_{0_{\alpha }}\cdot R/E_{a_{\alpha }}\cdot g\left(\alpha \right)\right)-E_{a}/RT_{\alpha ,i}$$

Then, plotting $\ln\left(\beta_{i}/T_{\alpha ,i}^{2}\right)\;\mathrm{vs.}\;1/T_{\alpha ,i}$ allows the obtention of activation energy from the slope. 

#### 2.7.3. Flynn–Wall–Ozawa (FWO) Method

The FWO method [15,16] used the Doyle approximation (Equation (11)) [24] to simplify the integral temperature in Equation (7). The conversion function is denoted by the symbol g(α), and the subscripts i and α are related to the heating rate and conversion values, respectively. The activation energy can be calculated by equating the slope of the graph (ln (β) vs. 1/T) to $1.052\;E_{\alpha }/R$.

(11) $$\left(\beta_{i}\right)=\ln\left(A_{0_{\alpha }}\cdot E_{a_{\alpha }}/R\cdot g\left(\alpha \right)\right)-5331-1052E_{a}/RT$$

(12) $$g\left(\alpha \right)=\left(A_{0}/\beta \right)\cdot 0.00484\cdot e^{-1.052\cdot E_{a}/RT}$$

#### 2.7.4. Friedmann Method

The method proposed by Friedman [17] uses the derivative of the conversion (Equation (13)) so that Ea calculated is a precise value, as it does not use any mathematical approximation for the integral temperature. However, because it is a differential method, its accuracy is limited by signal noise. The activation energy can be calculated from the slope of a graph (ln (β·dα/dt) vs. 1/T).

(13) $$\ln\left(d\alpha /dt\right)\equiv \ln\left(\beta_{i}\cdot d\alpha /dt\right)=\ln\left(A\cdot f\left(\alpha \right)\right)-E_{a}/RT_{\alpha ,i}$$

### 2.8. Master Plots and Compensation Effect

The master plots determine those biomass reaction models where decomposition occurs in a single stage; i.e., the variation in Ea is not more significant than 20% concerning the average. The master plots considered the method suggested by ICTAC, following Equation (14) [25].

(14) $$Z\left(\alpha \right)=f\left(\alpha \right)g\left(\alpha \right)={\left(\frac{d\alpha }{dt}\right)}_{\alpha }T_{\alpha }^{2}\left[\frac{\pi }{\beta T_{\alpha }}\right]$$

where the first term (f(α)g(α)) represents theoretical *Z*(*α*) values and the second term ${\left(\frac{d\alpha }{dt}\right)}_{\alpha }T_{\alpha }^{2}\left[\frac{\pi }{\beta T_{\alpha }}\right]$ represents experimental values. It considers that the second experimental factor had no significant effect on the results. The *Z*(*α*) values contemplate half conversions for better presentation. The pre-exponential factor was calculated from the model-free method proposed by Vyazovkin through the compensation effect following Equation (15) [20]. The Log *A_i_* and *E_i_* pairs were calculated for 12 functions as reported in the Supplementary Materials (Table S1), which were replaced by *g*(*α*) in Equation (16) [26].

(15) $$\log A_{i}=\alpha E_{i}+b$$

(16) $$\ln\left[\frac{g_{i}\left(\alpha \right)}{T^{2}}\right]=\ln\left[\left(\frac{A_{i}R}{\beta E_{i}}\right)\left(1-\frac{2R\bar{T}}{E_{i}}\right)\right]-\frac{E_{i}}{RT}$$

where a and b are compensation parameters and the subscript *i* corresponds to each conversion function used for the calculation of each pair Log *A_i_* and *E_i_* and $\bar{T}$ corresponds to the experimental mean temperature.

### 2.9. Pyrolysis Tests

A lab-scale stainless steel reactor (60 mL) heated by an electric temperature-controlled furnace provided relevant data from the pyrolysis tests (N_2_, 60 mL·min^−1^, biomass, 2 g). The temperature program was as follows: heating (5 °C·min^−1^) from 35° up to 350 °C and then isothermal for 30 min. The kinetic analysis of TGA assays provides the final pyrolysis temperature. The lower heating rate used here allowed the collection of more samples during the pyrolysis tests to ensure proper characterisation of the evolved gaseous stream by gas chromatography. A 50 mL balloon immersed in an ice-water bath collected the condensable pyrolysis compounds. The condensable fraction was also analysed by GC/MS.

On the other hand, the permanent gases passed through particle and silica-gel traps before GC-TCD analysis. Next, the pyrolytic fractions were gravimetrically quantified, considering residues deposited into system connections, and the gas fraction by difference. Finally, the experimental system was washed with acetone (Merck, MS grade) in an ultrasonic bath for 30 min before and after assays. 

### 2.10. Characterisation of Bio-Oils by GC/MS

A GC/MS (Shimadzu, QP2010 plus) analysed the chemical composition of condensable fractions using vials containing one microliter of bio-oil into a millilitre of acetone (Merck, GC grade). The GC configuration was as follows: Capillary column Rtx-5 ms (30 µm, 0.32 mm, 0.32 µm), Injector Temperature, Ion source, and Transfer line (250 °C), Oven temperature program (starting at 35 °C, then up to 180 °C (5 °C·min^−1^), and finally up to 300 °C (20 °C·min^−1^), sample volume 1 µL injected in Split mode (5.0 ratio), with Helium G6.0 (Indura, 99.9999%) as the carrier gas (Pressure control mode at 10 kPa). The MS unit operated in electron impact mode at 70 eV and SCAN mode (m/z: 2~500). The area normalisation method provided the detected species relative areas.

### 2.11. Characterisation of Permanent Gases by GC-TCD

The permanent combustion gases were analysed by gas chromatography using a thermal conductivity detector (GC-TCD) in a Shimadzu apparatus (GC-2014). Samples were injected in an injector (packed type, 80 °C) coupled to a packed column (Supelco 60–80 Carboxen 1000, 15 ft), using ultra-pure Nitrogen (Indura, 99.999%) as carrier gas (65 mL·min^−1^) in an isothermal programmed oven at 80 °C for 15 min, and finally conducted to TCD (100 mA, 100 °C). The primary gaseous species detected were hydrogen, oxygen, carbon monoxide, methane, and carbon dioxide. Species were identified and quantified by comparing retention times and calibration curves obtained from previous runs using standard gases. 

### 2.12. Analytical Pyrolysis Tests (Py-GC/MS)

The Py-GC/Ms assays were carried out on a CDS Pyroprobe 5200 HPR Micro-pyrolizer, analysing the evolved gases using a Perkin Elmer Clarus 690 Gas Chromatograph connected to a Perkin Elmer Clarus SQ-8T MS Detector Mass Spectrometer. Five milligrams of the sample were placed in the microreactor and heated to 350, 450, and 550 °C in an inert N_2_ atmosphere. A heating rate of 30 °C·min^−1^ and 60 s of pyrolysis was used for slow pyrolysis, while 10,000 °C·s^−1^ and 15 s were used for fast pyrolysis, respectively. Comparison of experimentally obtained ionisation patterns with those listed in the National Institute of Standards and Technology Library (NITS) allowed the chemical compound identification.

## 3. Results and Discussion

### 3.1. Characterisation of Biomass Samples

Table 1 summarises the characterisation results (proximal analysis and calorific value) for investigated biomass samples. Proximal analysis was conducted in triplicate, using samples (as received) and reporting data considering dry basis and averaged values. No significant differences among species were observed in volatile material (82~86%) and fixed carbon (12.6~16%) contents. The ash content, on the other hand, showed dispersion, with NO and EC samples having the highest values, followed by PR, ruling out the possibility that the amount of ash depends on the type of wood. It is critical to consider the ash content in thermochemical biomass conversion, particularly pyrolysis, because it has catalytic effects in forming compounds [27]. Table 1 also shows registered calorific values of samples (as received and previously oven-dried). As expected, the dry samples had a higher calorific value, but the difference was not as pronounced in samples with lower moisture content, such as EN.

Chemical composition is one of the primary distinctions between hardwoods and softwoods (See Table 2). The holocellulose content of the softwoods represented here by PR was like that of NO, a hardwood, and lower than that of other hardwoods. Holocellulose generally comprises equal parts hemicellulose and cellulose in most trees [28]. However, hemicellulose is found in higher proportions in EN and EC as they are fast-growing trees that need to produce more hemicellulose than other polymers. As a result of the high content of hemicellulose formed mainly by mannose monomers rather than lignin, such species might sometimes be considered semi-hard, mainly if they are felled at an early age [28,29]. Similarly, PR and NO had a higher lignin content than the other samples, but the difference was that because PR is a softwood, the lignin is primarily composed of guayacil units, whereas NO is also composed of syringil [28]. These properties are critical for biomass thermal behaviour in thermochemical conversion processes since they determine the products’ chemical composition.

### 3.2. Effect of the Heating Rate

**Figure 2:**
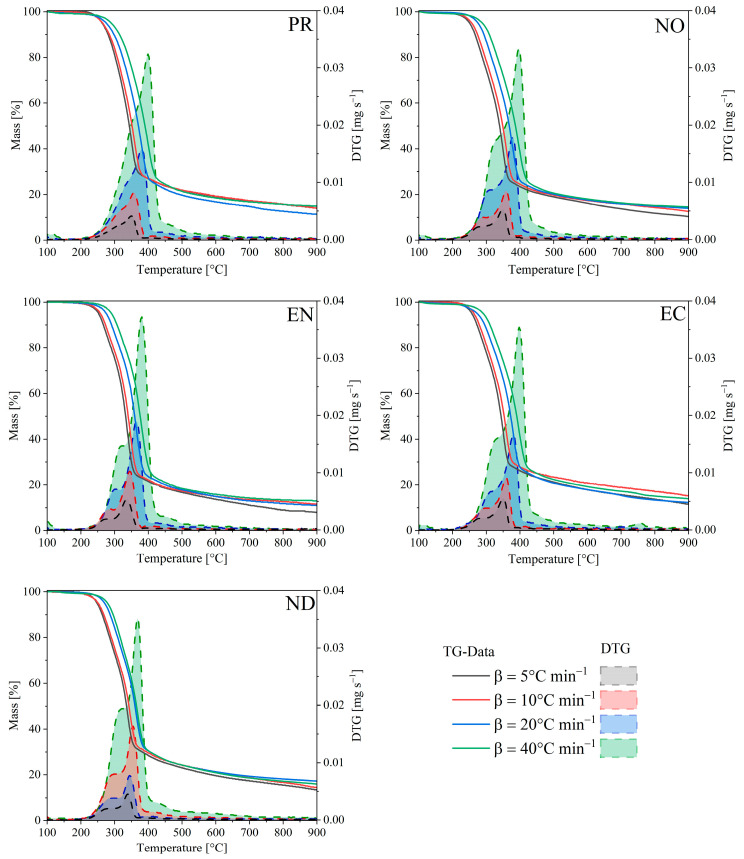
TG and DTG profiles for biomass samples at different heating rates.

Figure 2 shows the thermograms (TG) and their derivative (DTG) for the biomass samples at different heating rates. The DTG profiles show two overlapping peaks corresponding to hemicelluloses and cellulose (in the active pyrolysis zone) and a tailing zone associated with lignin decomposition (in the passive pyrolysis zone). Thus, the lignocellulosic pyrolysis processes (excluding drying) consider three stages, with starting and ending temperatures influenced by the heating rate. The first stage (200~340 °C) corresponds to the hemicellulose decomposition, and the second stage (300~450 °C) considers the cellulose decomposition. The literature indicates that lignin has a broader temperature range (180~900 °C) [30], covering active and passive pyrolysis. As reported elsewhere, the tailing zone begins once the cellulose has already reacted, showing a lower reaction rate attributable to the remanent lignin degradation [31]. 

As a result of increasing the heating rate (from 5 to 40 °C·min^−1^), a displacement of the temperature peaks and the DTG curves towards higher values without modifying the thermal profiles. From a kinetic point of view, this thermal behaviour suggests that the reaction rate depends only on temperature. Such behaviour confirms that an increase in DTG curve amplitude is a consequence of the rising heating rate. Furthermore, the observed pyrolytic cracking mechanism is independent of the heating rates under the explored experimental conditions. The residence time could be responsible for this lateral shift or degradation delay. Then, the sample must reach higher temperatures at higher heating rates to have enough time to complete decomposition. As the heating rate increases, a progressive mass loss occurs, leading to higher volatile material production. Variations in the maximum mass loss rate are associated with heterogeneous structure and multicomponent biomass composition. Each component has an individual decomposition behaviour, reaching maximum reaction rate at defined temperature ranges. However, some components can degrade simultaneously at higher heating rates, and adjacent peaks can coalesce to form prominent, overlapping peaks. For example, softwoods such as pinus (radiata, pinaster, and sylvestris) showed overlapped peaks associated with hemicellulose and cellulose [31,32]. On the other hand, hardwood species showed resolute DTG peaks. 

The values of T_onset_ marked the starting point for the thermal decomposition of less thermally stable components (Table 3). At around 200 °C, volatiles began to be produced in the biomass species, with the ND having the lowest T_onset_ (172 °C) and the highest reactivity (17.3%). T_onset_ and T_offset_ varied with heating rate, but the differences in T_onset_ of NO samples were insignificant. As the hardest wood of the tested samples, their highly dense cell walls prevented heat transfer to the particle core, slowing thermal decomposition. This effect was reflected in a significant increase in the T_offset_ of NO compared to other samples because it required a higher temperature (translated into a longer residence time here) to achieve complete lignin degradation. Despite this, EN had the lowest reactivity, with values no higher than 12.8%, such as heated samples at lower rates, confirming an intrinsic dependence on temperature and wood type. 

### 3.3. Kinetic Analysis

**Figure 3:**
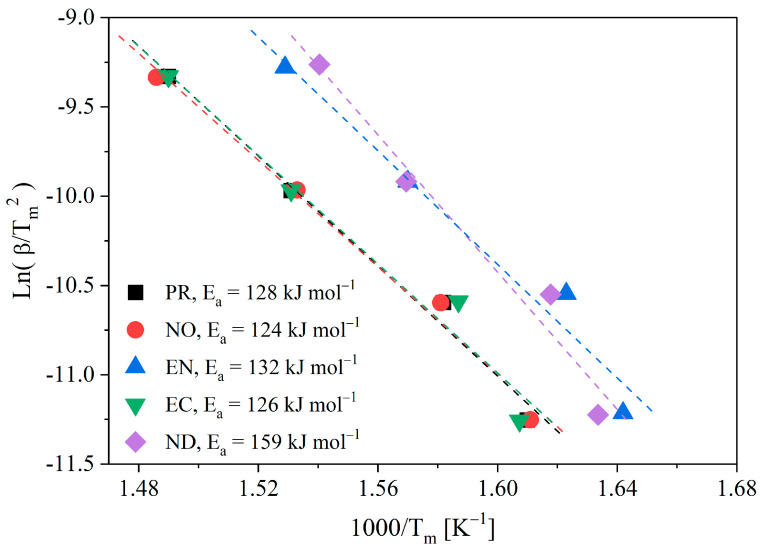
Kissinger plot for pyrolysis biomass.

Using registered data, Kissinger and isoconversional models (KAS, FWO, and FR) provide biomass kinetic parameters. Firstly, Ea and A_0_ were calculated from the Kissinger method (Equation (8)). Figure 3 shows the Kissinger graphs for evaluated biomasses, and Table S2 summarises the values of Ea and $A_{0}$ obtained from correlations.

The Kissinger method affords an approximated estimation of kinetic parameters considering that $f’\left(\alpha_{m}\right)$ must be independent of the heating rate. Thus, the first term on the right side of Equation (8) could not be constant and the registered data might deviate from a straight line, introducing a systematic error in parameter calculation. The independence of $f’\left(\alpha_{m}\right)$ with β is only valid for the first-order reaction model since f(α)=(1−α) and f’(α)=−1. A Kissinger method limitation is that the obtained activation energy value does not consider process kinetic complexity. Moreover, the Kissinger method adequately represents simple reaction kinetics, as considered by Equations (1) and (3), obtained from the maximum observed reaction rate. However, biomass species show a higher dependence on activation energy with conversion. Unfortunately, such variations are typical in biomass samples, and free model methods (based on temperature peaks) cannot detect them. The Ea values calculated for the various Chilean wood species ranged from 124 to 160 kJ·mol^−1^, consistent with Ea values calculated by the Kissinger method for sawdust from wood mixtures ranging from 145 [33] to 160 kJ·mol^−1^ [34]. Unlike Ea, the value of the pre-exponential factor varies dramatically across different types of biomasses, which may reflect the error induced by calculating Ea if the process occurs in one step [33]. This effect reflected the need to perform the conversion calculations and compare them with the Kissinger method.

### 3.4. Activation Energy Evaluation Using Isoconversional Methods

Table 4 summarises the average activation energies obtained by isoconversional methods (KAS, FWO, and FR). A comparative analysis (F-test) determined whether there were significant differences in the average activation energies obtained from the various methods. The FR method yielded higher average activation energy values (except for the PR sample), demonstrating significant differences from the KAS method for the pyrolysis of the NO, EN, EC, and ND samples. According to the KAS method, all the biomass showed thermal decomposition with equal activation energy, implying that the decomposition is independent of the chemical composition of the wood under the study conditions. After applying the three methods for pyrolysis of PR, the obtained values showed homogeneity; however, the opposite occurred for ND, which showed significant differences in Ea calculated by the three methods. When not considering the importance of the reaction stages, a global chemical decomposition leads to a discrepancy between the average values of the activation energies, leading to errors when interpreting the results.

**Figure 4:**
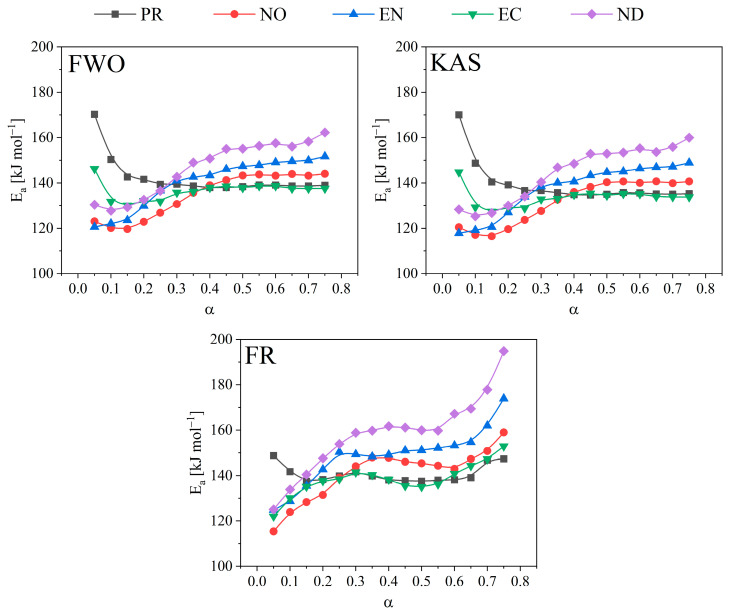
Activation energy dependence on conversion using FWO, KAS, and FR methods.

The Arrhenius plot of the isoconversional methods (KAS, FWO, and FR) and the summary table for Ea and correlation coefficients (R^2^) are shown in Figures S1 and S2 of the Supplementary Materials to help explain the variations in the thermal behaviour of the biomass samples. These display the conversions assessed between 0.05 and 0.75 with high correlation coefficients (R^2^ > 0.960) attained. The calculated correlation coefficients for conversions 0.8 are less than 0.85 and were not considered. The kinetic data fitted to the Friedman model, regarded as one of the most accurate isoconversional approaches, provides confidence to Ea calculated values. Figure 4 shows the activation energy tendencies as a function of conversion. Tables S3–S7 in Supplementary Materials detail the Ea values derived using the isoconversional methods for each biomass and their respective correlation coefficients. 

An evident variation in the activation energy at the evaluated conversion range for the biomass species was observed after using the three methods, mainly for the EN and ND species. The obtained Ea variation is a result of biomass heterogeneity due to polymer fractions with volatilisation characteristics, and according to Vyazovkin [20], considering Ea as a variant when the difference between the upper and lower value is higher than 20% concerning the average Ea. Thus, as decomposition progresses, the polymerisation degree of pyrolytic reactive compounds could influence the activation energy. For PR, significant differences were registered using the FWO and KAS (Table S3) methods with high energies to reach a conversion of 0.05, the same that decreased for higher conversions and remained practically constant from 0.4 conversions. On the other hand, the variation in E_a_ using the FR method was 8%, implying that the thermoconversion occurred in two scenarios, single-step or multi-step. The Ea determined by the three techniques had somewhat higher values at low PR conversions (0.05) (KAS and FWO = 170 kJ·mol^−1^, FR = 148 kJ·mol^−1^) that declined as the conversion rose to 0.25, stabilised, and essentially stayed constant up to the high conversions (0.75). Such results are related to PR as a soft wood with a lot of lignin in the intercellular walls, which shields the cellulose and hemicellulose [29]. To leave the hemicellulose and cellulose unprotected, so that they might later decay, some lignin linkages must be broken in the first reaction, mainly an interpolymeric bond cleavage reaction. Lignin tends to melt at temperatures above 300 °C, so the general decomposition Ea is relatively low. However, it also indicates a rise in activation energy to get rid of it before it causes repolymerizations and encourages char development [35]. 

The calculated Ea for hardwoods begins with relatively low values (120 kJ·mol^−1^) at low conversions and gradually rises as conversion increases, a definite indicator of sequential breakdown from a less stable polymer such as hemicellulose to lignin. For NO and EC, the variation in Ea was less than 20% when using the FWO and KAS methods, so it is considered a single-step decomposition, but with FR, the variation in Ea was higher and is considered multiple-step [20]. The observed activation energy differences using the FWO, KAS, and FR methods are also due to their intrinsic characteristics. Differential methods (e.g., FR) use the global reaction rate values, while integral methods (FWO and KAS) describe the system evolution, introducing a systematic error when the activation energy varies significantly with the conversion [25]. Then, values obtained using the FR method are more reliable than those obtained by FWO and KAS [36]. One of the disadvantages of the isoconversional method is related to determining the A_0_ and the reaction order. Therefore, it is necessary to apply complementary methods to safely estimate these parameters of the kinetic triplet, as described in the following sections.

### 3.5. Master Plot

**Figure 5:**
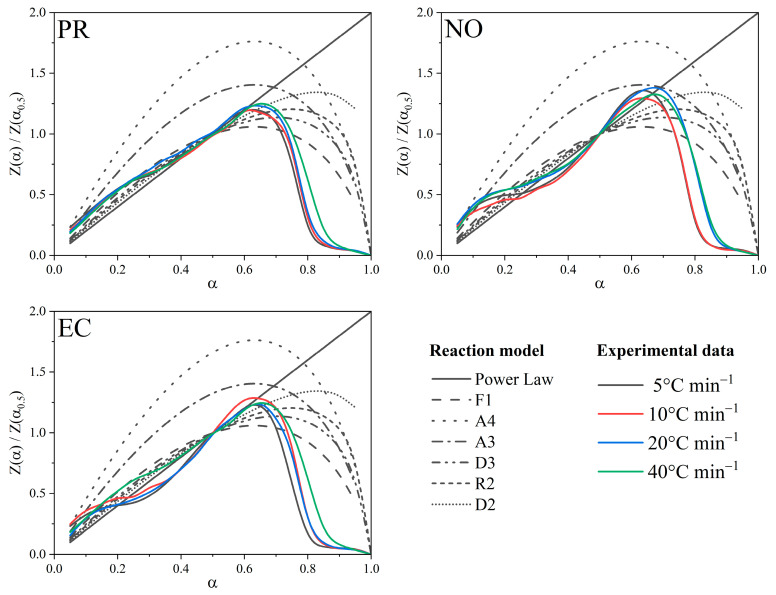
Master plot of theoretical and experimental Z normalised to 0.5 conversions for PR, NO, and EC samples.

Since all three isoconversional methods were fit to the experimental data, testable methods must establish the reaction model. For the PR, NO, and EC samples, where at least one of the models was allowed if the reaction occurred in a single step, master plots were performed to identify the conversion models that best describe the biomass decomposition (Figure 5). For the conversions range (0.05 to 0.35), the F1 model accurately describes the PR decomposition behaviour and, then, a mixture of all models (except A3 and A4) where the power law stands out; however, the proposed models fit the final conversion part (alpha higher than 0.65). The same effect occurs on the NO and EC samples, where, in addition, the first phases of the decomposition fit different models at different conversion values, confirming multi-steps of thermochemical decomposition [25,37]. Such behaviour is prevalent with high ash-content biomasses, which can generate abrupt changes in the chemical reactions on the surface of the char formed. The biochar resulting from the pyrolysis is self-doped with the ashes present in the biomass, which provides a catalytic activity that can generate potential chemical applications; however, for the case study of the decomposition kinetics, it complexes the system [38]. The ash content, like Ea, affects the reaction rate represented by the pre-exponential factor. Because this is a multi-step process, it is convenient to calculate A_0_ concerning the activation energy through the compensation effect.

### 3.6. Compensation Effect

**Figure 6:**
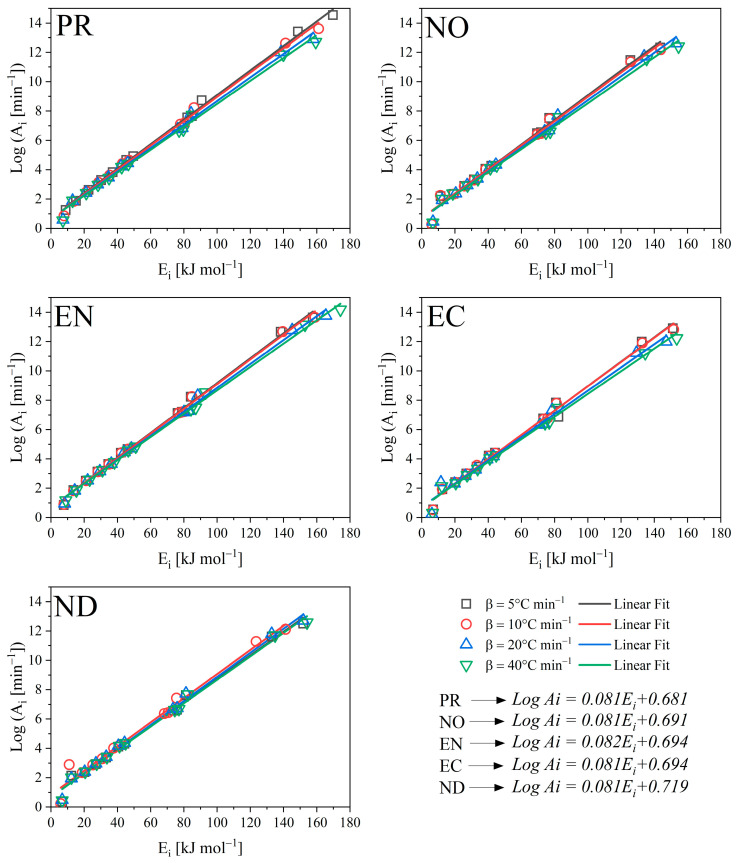
Compensation effect for the estimation of the pre-exponential factor.

Because isoconversional methods calculate model-free Ea, it is simple to calculate the pre-exponential factor from model-free techniques such as the compensation effect. Log A_i_ and E_i_ pairs were calculated from 12 conversion functions as indicated in the methodology. Thus, Figure 6 shows their linear regressions. For tested heating rates and biomasses, all Log Ai and Ei pairs fit a straight line with a high degree of correlation (0.99). According to Vyazovkin [20], the high regression value enables the computation of an appropriate pre-exponential factor using the compensation line as a function of Ea determined using an isoconversional approach. Using the line equation reduces the systematic error caused by the oscillation produced by couples that do not strictly fall on the line. Even though the pairs were estimated at various heating rates, the linear regressions stacked one on top of the other, yielding a single compensation line for each biomass. In this sense, regardless of the conversion function used, the pre-exponential factor would be a function of the conversion and the temperature at which the conversion occurs.

Despite the differing biomasses, the compensation line equations were relatively similar; nonetheless, these minor changes were apparent when computing the rate constant. For this reason, it is not advisable to unify the equations of the compensation line when dealing with different biomasses. Instead of Ea values as close to the compensation line values as possible, it confirms that decomposition occurred similarly in all the woods studied under three main models: three-dimensional diffusion, one-dimensional and with contributions of less than the first order model of Mampel [39], and contracting sphere.

After calculating the pre-exponential factor from the compensating effect, the reaction constant comes from replacing the A_α_ and E_α_ calculated for each conversion to which a temperature corresponded (Table 5). Because of the minimal changes caused by Ea determined using the FR method, A_0_ values for PR were in a small range (7.04 × 10^11^–5.83 × 10^12^ min^−1^). NO (1.20 × 10^10^–4.26 × 10^13^ min^−1^) and EC (3.33 × 10^10^–1.02 × 10^13^ min^−1^) showed modest oscillations but higher than PR, generated by fluctuations in Ea in the same way. Finally, the changes in A_0_ were more pronounced for EN (9.85 × 10^10^–1.10 × 10^15^ min^−1^) and ND (7.42 × 10^10^–3.35 × 10^16^ min^−1^), owing to higher resistance to heat breakdown. Such results were due to the unique qualities of the wood as well as the likely availability of the lignocellulosic material, which might delay the release of volatiles from within the particle and result in more thermally stable repolymerization. For biomasses such as bamboo, A_0_ values ranged from 1.85 × 10^−4^ to 1.44 × 10^−7^ (s^−1^) for Ea 42.48–165.45 kJ·mol^−1^, as reported elsewhere [38], implying substantially slower breakdown processes than for the woods evaluated in this study. However, similar A_0_ (5.96 × 10^9^ s^−1^) and Ea (146.22 kJ·mol^−1^) values have been reported elsewhere [40], using model-free approaches for the thermal decomposition of maple waste leaves, which might have a significant potential for generating value-added chemicals. 

**Figure 7:**
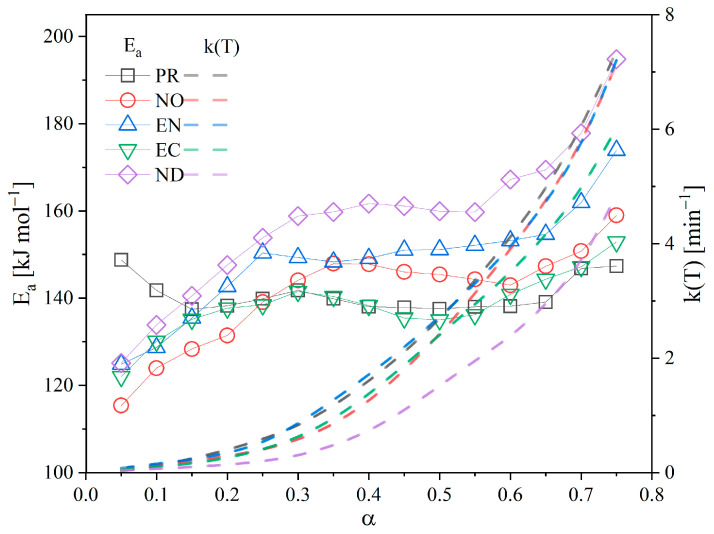
Activation energy and reaction kinetic constant concerning conversion.

Figure 7 depicts the Ea and k(T) relationship for each conversion (k as a function of T_α_). Then, the wood with the highest Ea (in this case, ND) has the higher resistance to heat decomposition, and its process was the least accelerated of investigated biomasses. This assumption, however, cannot be made just based on the Ea value because EC had one of the lowest Ea values, but its decomposition was the second slowest. In addition to being highly dense, hardwoods such as EC contain significant amounts of fixed carbon that resist volatilisation and a low proportion of volatile matter. The converse happened with EN, which as a hardwood, poses a relatively low initial Ea, allowing a quick release of volatile materials, which functioned as a self-promoter for decomposing the remaining organic portion. As a result, the decomposition of EN was greatly accelerated, with behaviour remarkably comparable to that of PR, a softwood with reduced resistance to decomposition (lower Ea) and the fastest of the study group. Thus, these biomasses (mainly PR and EN) might be suitable for conversion into value-added products.

However, despite the different behaviours of each studied biomass, all decompositions were accelerated, as shown by the k(T) profile [20]. One of its distinguishing features is the high carbon yield (30%) of biomass degradation by pyrolysis procedures at modest heating rates. In addition, as the ash does not lose mass throughout the reaction, the biochar generated contains a high ash concentration due to the initial chemical composition of the wood. These rapidly adapt to the large surface area of the biochar generated and produce a catalytic action that promotes wood breakdown, aided by the gases from the volatilisation process [2]. As a result, several authors classify biomass pyrolysis as an initially endothermic but later exothermic and accelerated process [2,38].

### 3.7. Distribution of Pyrolysis Products

In slow pyrolysis operations, the char production is usually high, as in this case, where it ranged from 33% to 36% for all woods, with no significant variations across samples, as summarised in Table 6. However, there were substantial variations in the liquid fractions. PR, for example, had the lowest thermal decomposition resistance because it allowed for the quick release of volatiles and water, resulting in the maximum amount of liquid fraction (48%). However, the liquid fraction content was not related to Ea since ND (wood with higher Ea and slower decomposition) produces a high value of the liquid fraction. Therefore, wood properties influence the amount of produced liquid, showing the main differences for PR as softwood (which may have more water inside the cell wall), followed by ND as hardwood. Ash concentration can also impact gas formation, as seen for EC, a hardwood that was the second most decomposition-resistant. Furthermore, the ash catalytic effect impacted the chemical reaction, which hastened decomposition and resulted in a more significant gas fraction (38%). On the other hand, woods such as NO and EN might be attractive for conversion into value-added products because they can generate reasonable amounts of pyrolytic liquid (37% and 39%, respectively) without sacrificing the target biochar from slow pyrolysis.

#### 3.7.1. Chemical Composition of the Liquid Phase of Pyrolysis

**Figure 8:**
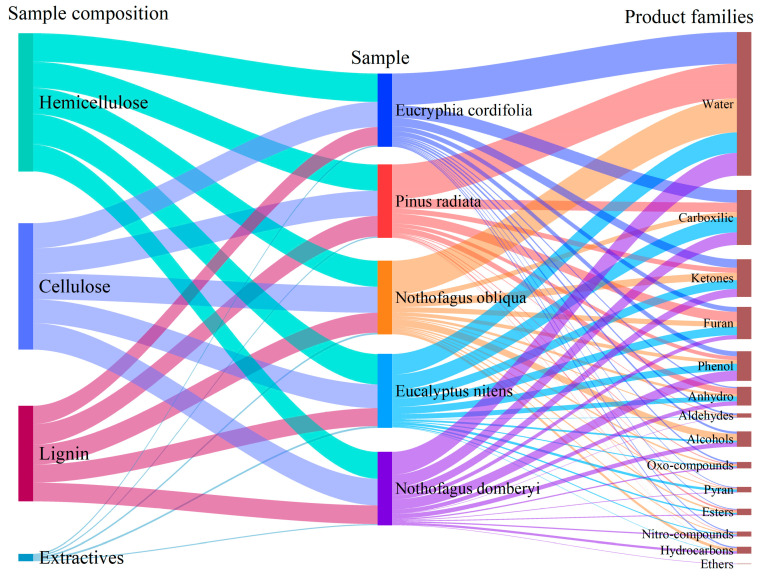
Distribution of liquid phase compounds concerning biomass chemical composition.

The bio-oil obtained from the thermochemical decomposition of cellulose, hemicellulose and lignin poses a complex composition of more than 200 species of diverse chemical nature. Figure 8 summarises the leading identified compounds families, including water, alcohols, aldehydes, anhydrosugars, carboxylic acids, ketones, esters, oxo-compounds, furan derivatives, hydrocarbons, phenolic derivatives, pyran derivatives, and ethers. Biomass pyrolysis begins at temperatures below 300 °C, implying a reduction in polymerisation, forming free radicals, removing water, and forming various compounds until a carbonaceous residue (biochar) is obtained [33]. Anhydrooligosaccharides, monomeric anhydrosugars, derivatives (primarily levoglucosan), furans, and cyclopentanones are the main products of depolymerisation. The fragmentation steps result in linear carbonyls such as aldehydes and aromatics such as phenols [34].

Slow biomass pyrolysis commonly leads to the primary compound formation (water and carboxylic acids) linked to the first stages of pyrolysis by the decomposition of hemicellulose. The EC, PR, and NO species produce more water during decomposition (43%, 47%, and 46%) than other tested biomasses. On the other hand, the species that produced the most carboxylic acids were EN, EC, and ND, with 20%, 17%, and 17%, respectively, which is consistent with the hemicellulose content, and as hardwood samples, the arrangement of the fibres encourages the formation of mainly acetic acids, as opposed to softwoods, which produces some acids of three to five carbons. The derivatives of furans and ketones, obtained by subsequent reactions involving the sugars liberated from cellulose [41] are other significant families. Furfural (7%) and 5-hydroxymethylfurfural (3%), found in the EN bio-oil, stood out among the furans. Because the samples underwent slow pyrolysis, most of the chemicals resulted from secondary reactions, which reduced the proportion of less stable molecules such as sugars. Of the anhydrous sugars found, some were 1,4:3,6-Dianhydro-α-D-glucopyranose; 2,3-Anhydro-D-galactosan; 1,3-Di-O-acetyl-α-β-D-ribopyranose at low concentrations (less than 1%); and 1,6-anhydro-β-D-Glucopyranose (Levoglucosan) was found up to 6% in obtained bio-oil from ND. Hemicellulose shows lower thermal stability and decomposes by dehydration (below 280 °C) and depolymerisation (at higher temperatures). Lignin is a heteropolymer macromolecule derived from three alcohol monomers (guaiacyl, syringyl, and p-hydroxyphenyl. Based on the cell wall predominant monomers, major plants groups have three main types of lignins: guaiacyl in gymnosperms (softwoods), guaiacyl–syringyl in angiosperms (hardwoods), and guaiacyl–syringyl–p-hydroxyphenyl in grasses [42,43]. The PR biomass with the highest lignin content produced fewer phenols (4%) than the other biomasses. In PR, part of the lignin had to degrade for the hemicellulose and cellulose to break down. The large percentage of furans (13%) and water obtained from PR pyrolysis confirmed the temperature effect on the produced phenolic rings at low temperatures and its final transformation into furans by secondary reactions releasing a substantial amount of water. Due to its complex structure, lignin is thermally more stable than hemicellulose and cellulose and produces more residual carbon [38]. Thus, lignin pyrolysis produced various phenolic products (syringol, methylsyringol, and methoxyeugenol) in hardwoods, reaching 14% in ND bio-oil and around 5~6% for NO and EN. The main phenolic compounds in PR were guaiacol, p-methyl guaiacol, and p-ethylguaicol.

#### 3.7.2. Evolution of Gaseous Species from Pyrolysis

**Figure 9:**
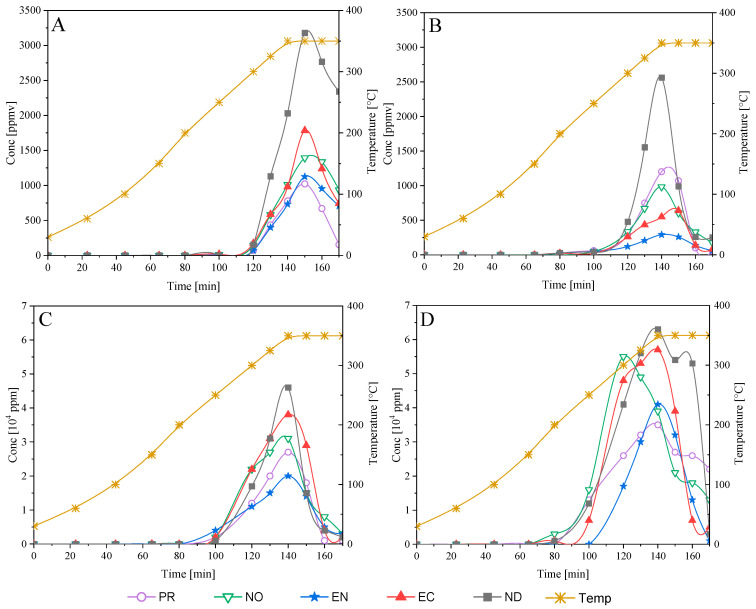
Permanent gases evolution from biomasses pyrolysis: (**A**): Methane; (**B**): Hydrogen; (**C**): Carbon monoxide; (**D**): Carbon dioxide.

Figure 9 shows the permanent gases (H_2_, CO, CH_4_, and CO_2_) evolution profiles evolved after condensation of the pyrolysis gaseous stream and measured by GC/TCD. Registered profiles show that degradation products appear when pyrolysis temperatures are closer to 200 °C. The maximum production of CH_4_ was registered at around 350 °C, reaching 3000 ppm for ND and concentrations between 1000 and 1800 ppm for the other samples. Furthermore, the hydrogen production was also higher in ND pyrolysis, reaching around 2500 ppm, while the other studied species did not exceed 1200 ppm. The CO and CO_2_ generation varies from 2000 to 5000 ppm and between 3500 and 6300 ppm, respectively. 

#### 3.7.3. Analytical Pyrolysis

**Figure 10:**
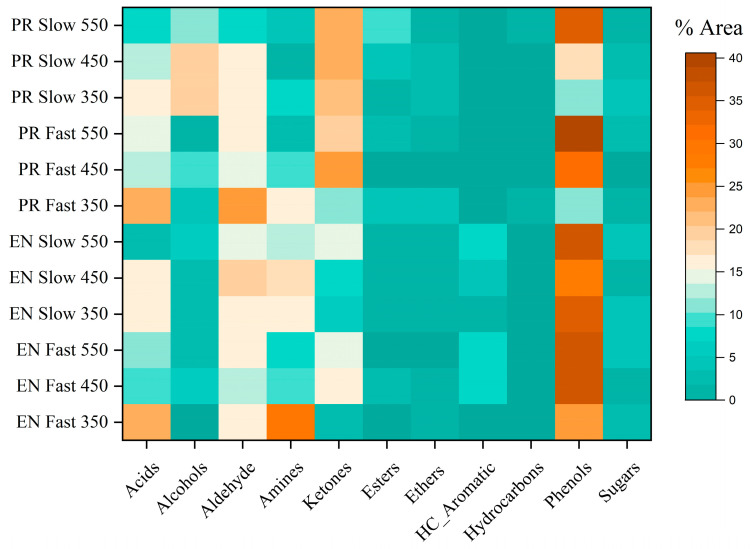
Chemical composition of condensable gases obtained from fast and slow analytical pyrolysis of EN and PR at different temperatures.

In addition to biochar, bio-oil is another primary pyrolysis product. Thus, using so-called fast pyrolysis increases the bio-oil yield and becomes more attractive to researchers nowadays. Then, the PR and EN samples (most prevalent biomasses in Chilean forestry production [11]) were tested in an analytical pyrolysis system (Py-GC/MS) due to the limitations of the lab-scale system for identification of chemical compositions of precursory gaseous compounds of bio-oils. Figure 10 and Table S10 (Supplementary Materials) show the registered results. The Py-GC/MS system accurately identified primary compounds formed during the pyrolysis (fast and slow), showing that phenolic groups were prevalent for the analysed biomasses. 

Furthermore, phenol concentrations depend on pyrolysis temperature and pyrolysis type (fast or slow). The PR sample poses a higher lignin content that decomposes over the entire pyrolysis range but with a higher conversion at high temperatures [2]. Thus, the phenol content (from PR samples) was higher in fast pyrolysis (40%) than the obtained by slow pyrolysis (34%) at 550 °C. However, the obtained bio-oil for both types of pyrolysis at 350 °C contains only 10% of phenols. For fast pyrolysis assays, compounds such as 2-methoxy-4-vinylphenol and cresol were the most prevalent components in this family, whereas, in slow pyrolysis, 2-methoxy-phenol and cresol were the main ones. Phenol, 2-methoxy- was the most representative compound at 350 °C in both fast and slow pyrolysis of PR, but not at higher temperatures. For EN samples, regardless of the kind of pyrolysis (fast or slow), the phenol concentration between 450 and 550 °C was approximately 36%, while at 350 °C, it ranged around 25–28%. For slow pyrolysis, compounds such as 2,6-dimethoxy-4-(prop-1-en-1-yl) phenol and 4-ethenyl-2,6-dimethoxy-phenol reported the highest concentrations, whereas, for fast pyrolysis, 2,6-dimethoxy-phenol and 4-ethenyl-2,6-dimethoxy-phenol were the phenol representatives. 

In contrast to the slow pyrolysis, the fast one produced a higher acid content (23% vs. 16%), which decreased as the temperature rose. Fast pyrolysis leads to rapid volatilisation, which prevents the formed acids from reacting again to form esters through secondary reactions [35]. A 3% of esters found in the slow pyrolysis and 0.6% in the fast pyrolysis supported such behaviour. The abundance of alcohols and ketones was another vital factor that did not necessarily depend on the pyrolysis conditions but on the biomasses. PR differs from EN in that it contains less holocellulose (cellulose and hemicellulose), but because of low decomposition resistance (given by the Ea values), these fractions were able to break down more quickly, resulting in four times more alcohol content and two times more ketones than EN. This results in a quick release of gases in a micropyrolysis system because the compounds’ short residence times prevent their subsequent decomposition.

The lab-scale system reported less than 2% of nitrogenous compounds. In contrast, the amino compounds (nitrogenous compounds with the most relevant presence in analytical pyrolysis) ranged widely from 1 to 30%. Such a result shows the contribution of analytical pyrolysis and the importance of conducting this research to identify the compounds produced at different pyrolysis conditions.

## 4. Conclusions

The kinetic parameters derived from the three isoconversion approaches were similar and accurately described the solid-state reaction degradation mechanism. Due to the slight fluctuation in the Ea values, some techniques represented degradation in a single step; however, the generated master plot proved that any reaction model did not describe the decomposition final section. Given this scenario, experimental results showed that the five studied samples decomposed in more than one step, and the reaction was characterised by three-dimensional and one-dimensional diffusion models using the pre-exponential factor from the compensatory effect. The high adjustment value of the compensation line assures accurate calculation of the pre-exponential factor and afterwards determines the Arrhenius reaction constants, showing that all decompositions were accelerated, with PR being faster than ND. The calculated kinetic parameters agreed with those previously reported using similar methodologies and samples.

Regarding value-added compounds, the liquid fractions were rich in phenolic compounds, ketones, and furans with broad industrial applications. Thus, *Pinus radiata* and *Eucalyptus nitens* were the most prolific species for such purpose, obtaining the highest fractions of liquid, followed by *Nothofagus domberyi*. Therefore, it is crucial to consider using analytical pyrolysis systems to compare results with lab-scale systems to obtain relevant information on the composition of the condensable fraction produced during pyrolysis.

## Figures and Tables

**Table 1:** Proximate analysis and Calorific values.

Sample	Volatile Matter (wt.%)	Fixed Carbon (wt.%)	Ash (wt.%)	Moisture (wt.%)	GCV ^a^ (MJ·kg^−1^)	GCV ^b^ (MJ·kg^−1^)	GCV ^c^ (MJ·kg^−1^)
PR (*Pinus radiata*)	83.41	13.94	2.65	9.48	17.82	20.98	32.41
NO (*Nothofagus obliqua*)	85.74	12.62	3.30	8.12	17.89	20.72	24.93
EN (*Eucalyptus nitens*)	85.14	13.34	1.51	5.86	17.59	18.26	29.33
EC (*Eucryphia cordifolia*)	81.97	15.18	2.87	9.09	17.52	19.06	27.83
ND (*Nothofagus domberyi*)	82.53	16.08	1.40	11.38	16.04	18.85	24.44

^a^ As received. ^b^ Dried at 378 K for 24 h. ^c^ Biochar from pyrolysis at 623 K for 30 min.

**Table 2:** Compositional analysis of biomasses (dry basis).

Sample	Holocellulose (%)	Cellulose (%)	Extractives (%)	Lignin (%)	Hemicellulose (%) ^a^
PR	70.08	34.25	1.79	28.12	35.83
NO	70.93	35.38	2.43	27.10	35.55
EN	73.76	32.30	2.52	24.49	41.46
EC	73.01	34.14	1.86	25.03	38.87
ND	72.82	36.13	1.45	25.39	36.69

^a^ Calculated by difference.

**Table 3:** Comparison of degradation parameters at different heating rates.

Sample	Heating Rate (°C·min^−1^)	T_onset_ (°C)	T_1_ (°C)	T_m_ (°C)	α_m_	DTG_m_ (mg·s^−1^)	T_offset_ (°C)	α_offset_	%R
PR	5	192	311	348	0.63	0.0041	386	0.83	13.9
	10	198	325	359	0.66	0.0085	403	0.85	14.5
	20	213	341	380	0.66	0.0158	426	0.86	10.7
	40	225	359	398	0.68	0.0324	452	0.89	14.2
NO	5	198	275	348	0.66	0.0053	384	0.84	8.8
	10	200	286	359	0.68	0.0090	398	0.86	10.1
	20	203	307	379	0.70	0.0179	434	0.89	13.7
	40	205	328	400	0.72	0.0329	448	0.90	14.7
EN	5	196	279	336	0.59	0.0055	386	0.82	7.6
	10	199	291	343	0.60	0.0105	395	0.88	10.7
	20	202	304	364	0.62	0.0189	428	0.88	10.7
	40	234	320	381	0.72	0.0372	438	0.91	12.8
EC	5	188	286	349	0.64	0.0056	383	0.82	11.1
	10	192	297	357	0.64	0.0092	405	0.86	13.1
	20	205	309	380	0.66	0.0167	429	0.86	12.1
	40	219	327	398	0.66	0.0354	446	0.87	13.6
ND	5	172	276	339	0.62	0.0046	374	0.80	13.5
	10	172	283	345	0.62	0.0080	394	0.81	17.3
	20	175	302	364	0.64	0.0164	417	0.85	16.4
	40	198	328	376	0.60	0.0346	436	0.85	15.0

**Table 4:** Averaged activation energies obtained from FWO, KAS, and FR methods.

Sample	Activation Energies, Ea ($kJ\cdot {mol}^{-1}$)		
	KAS Method	FWO Method	FR Method
PR	139.25	142.07	140.70
NO	131.60	134.70	140.92
EN	137.34	140.10	148.45
EC	133.50	136.56	138.38
ND	133.49	144.91	158.08

**Table 5:** Arrhenius kinetic parameters calculated by model-free methods for E_α_ (isoconversional FR) and A_α_ (compensation effect).

α	PR			NO			EN			EC			ND		
	E_α_ (kJ·mol^−1^)	A_α_ (min^−1^)	R^2^	E_α_ (kJ·mol^−1^)	A_α_ (min^−1^)	R^2^	E_α_ (kJ·mol^−1^)	A_α_ (min^−1^)	R^2^	E_α_ (kJ·mol^−1^)	A_α_ (min^−1^)	R^2^	E_α_ (kJ·mol^−1^)	A_α_ (min^−1^)	R^2^
0.05	148.76	5.83 × 10^12^	0.962	115.38	1.20 × 10^10^	0.982	124.85	9.85 × 10^10^	0.976	122.02	3.33 × 10^10^	0.957	125.12	7.42 × 10^10^	0.978
0.10	141.77	1.58 × 10^12^	0.982	123.96	6.01 × 10^10^	0.983	128.70	2.05 × 10^11^	0.986	130.10	1.49 × 10^11^	0.981	133.87	3.80 × 10^11^	0.981
0.15	137.46	7.04 × 10^11^	0.985	128.33	1.37 × 10^11^	0.988	135.45	7.37 × 10^11^	0.981	135.16	3.80 × 10^11^	0.980	140.51	1.31 × 10^12^	0.983
0.20	138.27	8.20 × 10^11^	0.986	131.46	2.45 × 10^11^	0.989	142.70	2.92 × 10^12^	0.979	137.70	6.09 × 10^11^	0.978	147.59	4.94 × 10^12^	0.980
0.25	139.87	1.11 × 10^12^	0.983	139.09	1.02 × 10^12^	0.989	150.36	1.25 × 10^13^	0.974	138.45	7.01 × 10^11^	0.975	153.86	1.59 × 10^13^	0.983
0.30	141.75	1.57 × 10^12^	0.985	144.08	2.61 × 10^12^	0.988	149.33	1.03 × 10^13^	0.976	141.51	1.24 × 10^12^	0.973	158.82	4.02 × 10^13^	0.983
0.35	139.87	1.11 × 10^12^	0.983	147.93	5.38 × 10^11^	0.990	148.30	8.47 × 10^12^	0.978	140.36	9.99 × 10^11^	0.974	159.76	4.79 × 10^13^	0.980
0.40	138.06	7.88 × 10^11^	0.986	147.79	5.23 × 10^12^	0.987	149.10	9.86 × 10^12^	0.977	138.31	6.83 × 10^11^	0.983	161.68	6.86 × 10^13^	0.984
0.45	137.83	7.54 × 10^11^	0.985	146.02	3.75 × 10^12^	0.986	151.01	1.42 × 10^13^	0.980	135.45	4.02 × 10^11^	0.986	161.13	6.19 × 10^13^	0.989
0.50	137.54	7.14 × 10^11^	0.986	145.43	3.36 × 10^12^	0.989	151.12	1.45 × 10^13^	0.984	135.05	3.73 × 10^11^	0.986	159.94	4.96 × 10^13^	0.989
0.55	137.99	7.78 × 10^11^	0.988	144.31	2.73 × 10^12^	0.992	152.14	1.75 × 10^13^	0.983	136.21	4.62 × 10^11^	0.986	159.73	4.77 × 10^13^	0.991
0.60	138.14	7.99 × 10^11^	0.992	142.97	2.12 × 10^12^	0.992	153.21	2.15 × 10^13^	0.983	140.88	1.10 × 10^12^	0.986	167.21	1.93 × 10^14^	0.991
0.65	139.12	9.61 × 10^11^	0.992	147.31	4.78 × 10^12^	0.994	154.66	2.83 × 10^13^	0.990	144.33	2.09 × 10^12^	0.985	169.43	2.92 × 10^14^	0.990
0.70	146.79	4.03 × 10^12^	0.999	150.78	9.17 × 10^12^	0.993	161.97	1.13 × 10^14^	0.976	147.23	3.57 × 10^12^	0.983	177.79	1.39 × 10^15^	0.989
0.75	147.34	4.47 × 10^12^	0.993	158.98	4.26 × 10^13^	0.989	173.92	1.10 × 10^15^	0.957	152.88	1.02 × 10^13^	0.982	194.81	3.35 × 10^16^	0.982
**Avg**	140.70	1.73 × 10^12^		140.92	5.54 × 10^12^		148.45	9.03 × 10^13^		138.38	1.53 × 10^12^		158.08	2.38 × 10^15^	

**Table 6:** Yields of biomass pyrolysis products.

Sample	Biochar (wt. (%))	Liquid (wt. (%))	Gas (wt. (%)) *
PR	33.95	48.06	17.99
NO	34.04	37.37	28.59
EN	35.78	38.83	25.39
EC	33.00	28.85	38.15
ND	31.11	41.93	26.96

* Values obtained by difference.

## Supplementary Materials

The following supporting information can be downloaded at: https://www.mdpi.com/article/10.3390/polym15122698/s1, Figure S1: FWO (left) and KAS (right) plots for biomass samples for different conversion values; Figure S2: FWO (left) and KAS (right) plots for biomass samples for different conversion values; Table S1: Different reaction models used for master plot construction and calculation of the compensation effect; Table S2: Kissinger kinetic parameters for biomass pyrolysis; Table S3: Activation energy and r-squared estimated by the three isoconversional methods (FWO, KAS, FR) for Pine (PR) decomposition; Table S4: Activation energy and r-squared estimated by the three isoconversional methods (FWO, KAS, FR) for Oak (NO) decomposition; Table S5: Activation energy and r-squared estimated by the three isoconversional methods (FWO, KAS, FR) for Eucalyptus (EN) decomposition; Table S6: Activation energy and r-squared estimated by the three isoconversional methods (FWO, KAS, FR) for Coigüe (ND) decomposition; Table S7: Activation energy and r-squared estimated by the three isoconversional methods (FWO, KAS, FR) for Ulmo (EC) decomposition; Table S8: Arrhenius reaction rate constants calculated for each biomass from A_α_ and E_α_ pairs; Table S9: Main pyrolytic products grouped by families (% Area) at lab-scale; Table S10: Main compounds identified in each family.
