# Supplementary material for: Pyrolysis of Chilean Southern Lignocellulosic Biomasses: Isoconversional Kinetics Analysis and Pyrolytic Products Distribution
Source: Polymers (Basel). 2023 Jun 16;15(12):2698. doi: 10.3390/polym15122698 (PMC10302438; doi:10.3390/polym15122698)
Supplement: Supplementary file 1 [file polymers-15-02698-s001.zip › polymers-2440471-supplementary.pdf]

polymers

MDPI

Supplementary Materials

Pyrolysis of Chilean Southern Lignocellulosic Biomasses: Isoconversional Kinetics Analysis and Pyrolytic Products Distribution

Cristian Cerda-Barrera [1], Kevin J. Fernández-Andrade [2,3] and Serguei Alejandro-Martín [2,3,*]

[1] Department of Industrial Processes, Universidad Católica de Temuco, 4780000 Temuco, Chile; ccerda@uct.cl
[2] Laboratory of Gas Chromatography and Analytical Pyrolysis, Universidad del Bío-Bío, 4030000 Concepción, Chile; kevin.fernandez2101@alumnos.ubiobio.cl
[3] Wood Engineering Department, Engineering Faculty, Universidad del Bío-Bío, 4030000 Concepción, Chile
* Correspondence: salejandro@ubiobio.cl

Supplementary Materials

Table S1. Different reaction models used for master plot construction and calculation of the compensation effect.

i	Title 2	Code	$f(\alpha)$	$g(\alpha)$
1	Power law	P4	$4\alpha^{3/4}$	$\alpha^{1/4}$
2	Power law	P3	$3\alpha^{2/3}$	$\alpha^{1/3}$
3	Power law	P2	$2\alpha^{1/2}$	$\alpha^{1/2}$
4	Power law	P2/3	$2/3\alpha^{-1/2}$	$\alpha^{3/2}$
5	One-dimensional diffusion	D1	$1/2\alpha^{-1}$	α^{2}
6	Mampel (first order)	F1	$1-\alpha$	$\ln(1-\alpha)$
7	Avrami-Erofeev	A4	$4(1-\alpha)[-\ln(1-\alpha)]^{3/4}$	$[-\ln(1-\alpha)]^{1/4}$
8	Avrami-Erofeev	A3	$3(1-\alpha)[-\ln(1-\alpha)]^{2/3}$	$[-\ln(1-\alpha)]^{1/3}$
9	Avrami-Erofeev	A2	$2(1-\alpha)[-\ln(1-\alpha)]^{1/2}$	$[-\ln(1-\alpha)]^{1/2}$
10	Three-dimensional diffusion	D3	$3/2(1-\alpha)^{2/3}[1-(1-\alpha)]^{-1}$	$\left[1-(1-\alpha)^{1/3}\right]^{2}$
11	Contracting sphere	R3	$3(1-\alpha)^{2/3}$	$1-(1-\alpha)^{1/3}$
12	Contracting cylinder	R2	$2(1-\alpha)^{1/2}$	$1-(1-\alpha)^{1/3}$
13	Two-dimensional diffusion	D2	$[-\ln(1-\alpha)]^{-1}$	$(1-\alpha)\ln(1-\alpha)+\alpha$

Table S2. Kissinger kinetic parameters for biomass pyrolysis.

Sample	E_a [kJ mol^{-1}]	A_0 [min^{-1}]	R^2
PR	128.02	1.28×10^{10}	0.993
NO	124.12	5.92×10^{6}	0.994
EN	132.27	5.59×10^{7}	0.983
EC	126.68	9.95×10^{6}	0.983

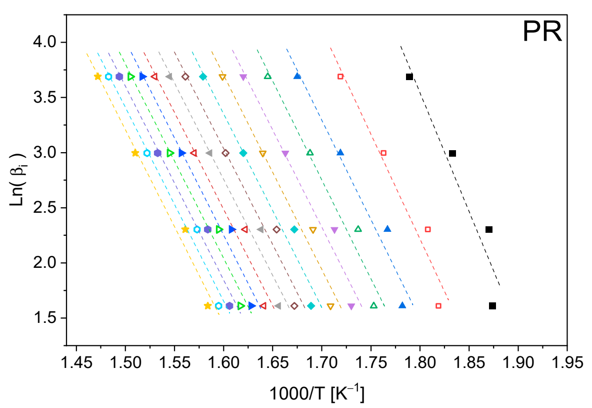
PR
Ln(β$_i$)
1000/T [K^{-1}]

(a)

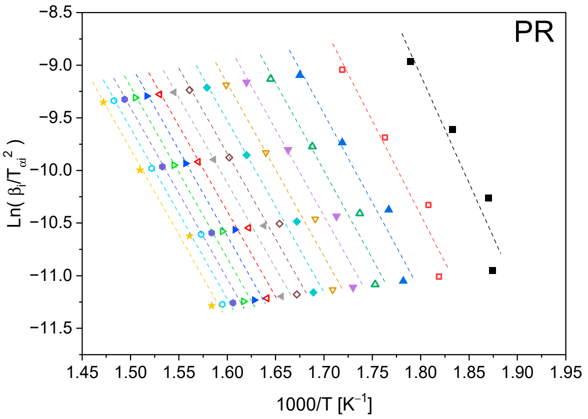
PR
Ln(β$_i$/T$_{\alpha i}$^{2})
1000/T [K^{-1}]

(b)

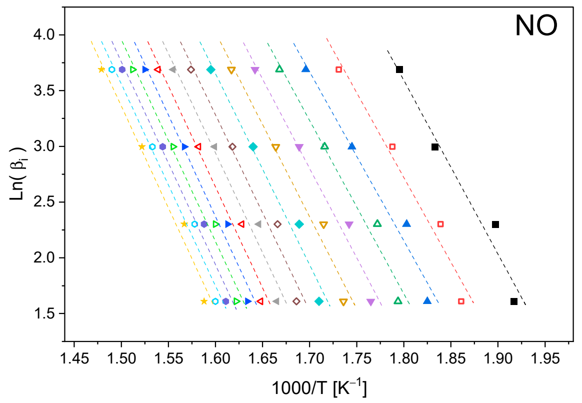
NO
Ln(β$_i$)
1000/T [K^{-1}]

(c)

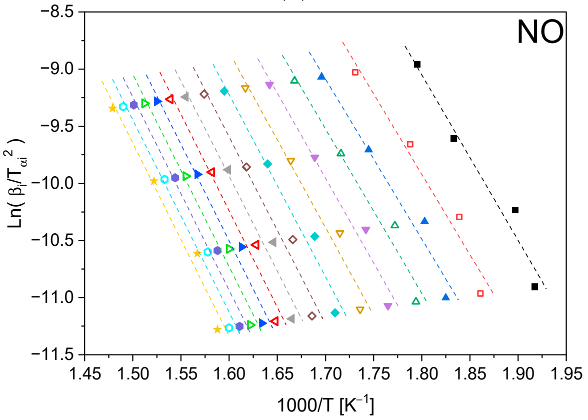
NO
Ln(β$_i$/T$_{\alpha i}$^{2})
1000/T [K^{-1}]

(d)

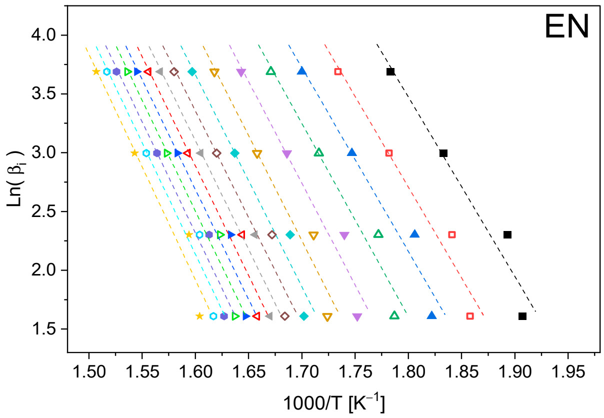
EN
Ln(β$_i$)
1000/T [K^{-1}]

(e)

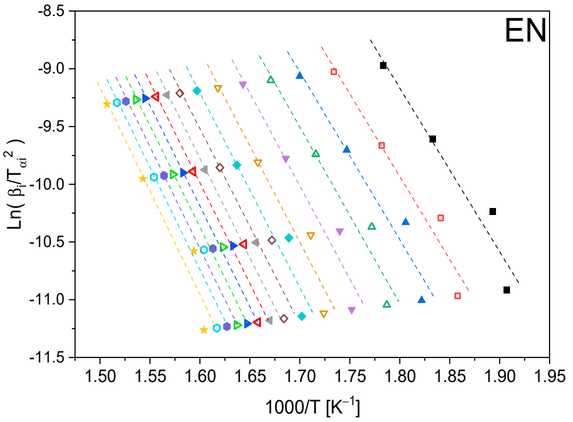
EN
Ln(β$_i$/T$_{\alpha i}$^{2})
1000/T [K^{-1}]

(f)

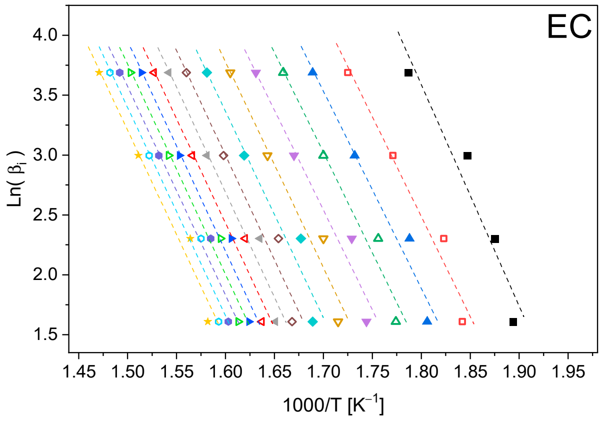
EC
Ln(β$_i$)
1000/T [K^{-1}]

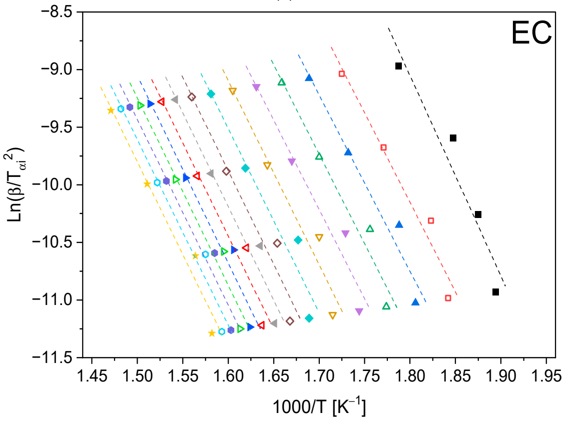
EC
Ln(β$_i$/T$_{\alpha i}$^{2})
1000/T [K^{-1}]

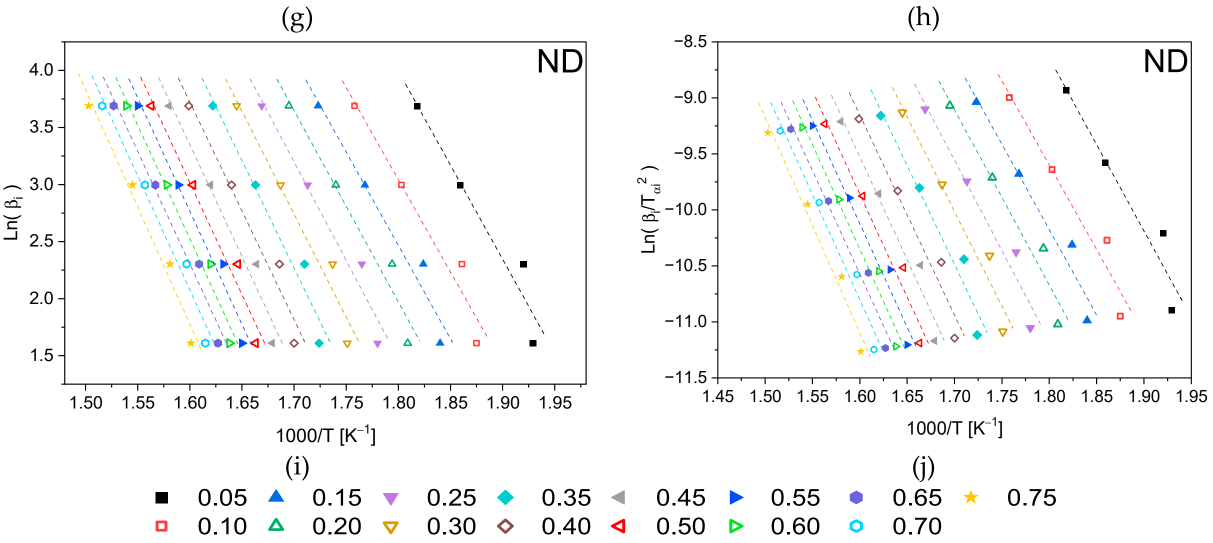

Figure S1. FWO (**left**) and KAS (**right**) plots for biomass samples for different conversion values.

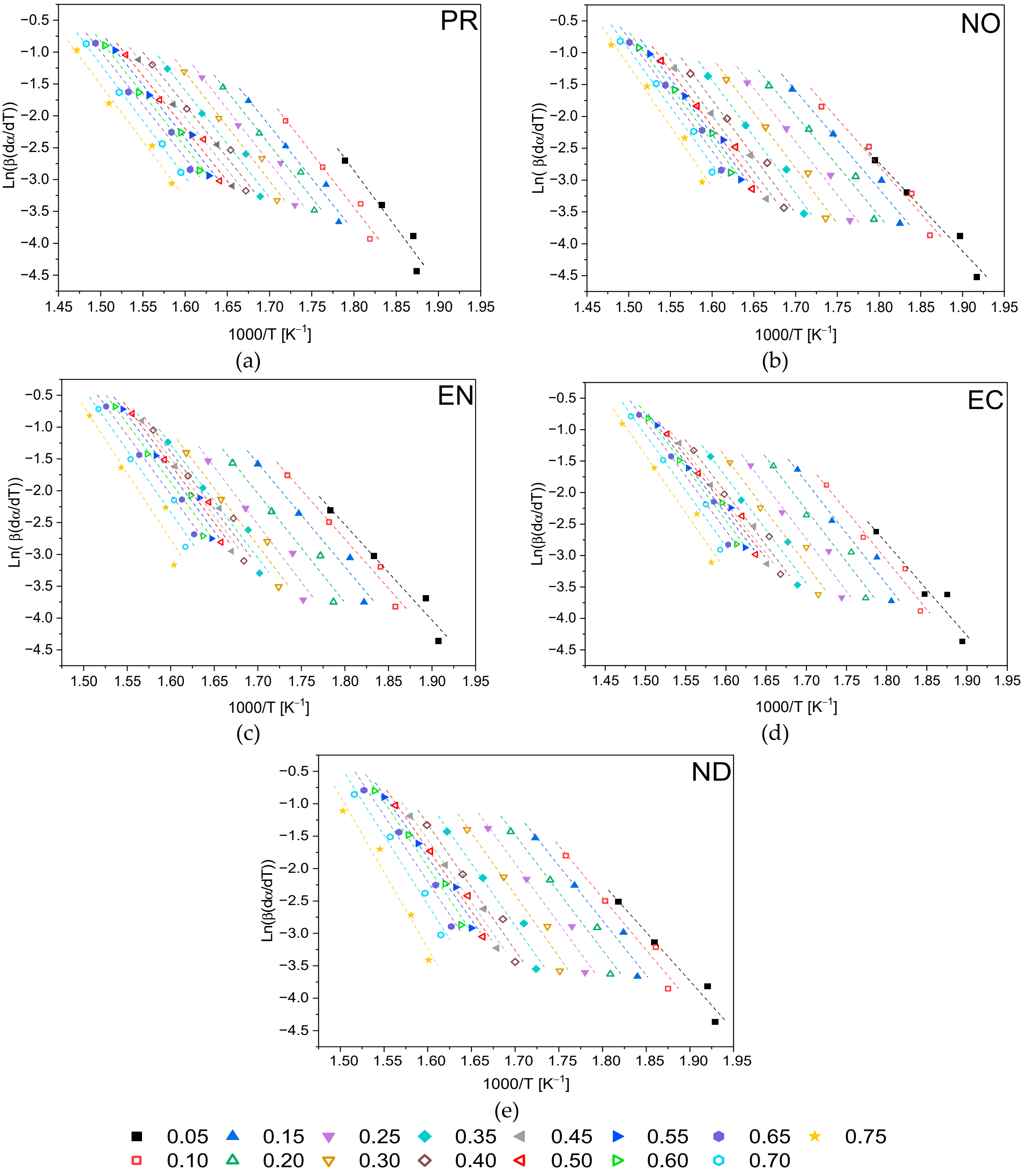

Figure S2. FWO (**left**) and KAS (**right**) plots for biomass samples for different conversion values.

Table S3. Activation energy and r-squared estimated by the three isoconversional methods (FWO, KAS, FR) for Pine (PR) decomposition.

α	FWO		KAS		FR	
	E_a [kJ mol^{-1}]	R^2	E_a [kJ mol^{-1}]	R^2	E_a [kJ mol^{-1}]	R^2
0.05	170.28	0.9527	170.05	0.9479	148.76	0.9622
0.10	150.36	0.9731	148.73	0.9700	141.77	0.9820
0.15	142.62	0.9801	140.43	0.9773	137.46	0.9854
0.20	141.61	0.9819	139.18	0.9794	138.27	0.9863
0.25	139.35	0.9832	136.64	0.9806	139.87	0.9833
0.30	139.46	0.9849	136.63	0.9826	141.75	0.9854
0.35	138.66	0.9833	135.70	0.9807	139.87	0.9831
0.40	137.94	0.9846	134.84	0.9822	138.06	0.9862
0.45	137.94	0.9846	134.69	0.9822	137.83	0.9854
0.50	138.34	0.9860	135.03	0.9838	137.54	0.9858
0.55	139.11	0.9875	135.76	0.9855	137.99	0.9882
0.60	139.06	0.9899	135.63	0.9882	138.14	0.9925
0.65	138.66	0.9897	135.15	0.9880	139.12	0.9922
0.70	138.67	0.9897	135.07	0.9880	146.79	0.9989
0.75	138.95	0.9908	135.28	0.9892	147.34	0.9928
**Average**	142.07		139.25		140.70	
**%Variation**	24.89	**Multi-Step**	23.22	**Multi-Step**	8.04	**Single-Step**

Table S4. Activation energy and r-squared estimated by the three isoconversional methods (FWO, KAS, FR) for Oak (NO) decomposition.

α	FWO		KAS		FR	
	E_a [kJ mol^{-1}]	R^2	E_a [kJ mol^{-1}]	R^2	E_a [kJ mol^{-1}]	R^2
0.05	123.06	0.9828	120.48	0.9802	115.38	0.98167
0.10	120.11	0.9833	117.09	0.9805	123.96	0.9830
0.15	119.68	0.9860	116.46	0.9836	128.33	0.9882
0.20	122.87	0.9866	119.66	0.9844	131.46	0.9887
0.25	126.84	0.9885	123.66	0.9865	139.09	0.9891
0.30	130.70	0.9867	127.62	0.9845	144.08	0.9883
0.35	135.63	0.9877	132.62	0.9857	147.93	0.9897
0.40	138.98	0.9870	136.01	0.9850	147.79	0.9867
0.45	141.25	0.9859	138.20	0.9837	146.02	0.9861
0.50	143.28	0.9877	140.31	0.9858	145.43	0.9890
0.55	143.72	0.9892	140.65	0.9875	144.31	0.9917
0.60	143.20	0.9901	140.02	0.9885	142.97	0.9923
0.65	143.92	0.9912	140.71	0.9898	147.31	0.9938
0.70	143.20	0.9901	139.90	0.9885	150.78	0.9926
0.75	144.04	0.9890	140.67	0.9872	158.98	0.9892
**Average**	134.70		131.60		140.92	
**%Variation**	18.09	**Single-Step**	18.43	**Single-Step**	30.94	**Multi-Step**

Table S5. Activation energy and r-squared estimated by the three isoconversional methods (FWO, KAS, FR) for Eucalyptus (EN) decomposition.

α	FWO		KAS		FR	
	E_a [kJ mol^{-1}]	R^2	E_a [kJ mol^{-1}]	R^2	E_a [kJ mol^{-1}]	R^2
0.05	120.59	0.9752	117.86	0.9714	124.85	0.9763
0.10	122.10	0.9801	119.16	0.9769	128.70	0.9858
0.15	123.54	0.9790	120.54	0.9757	135.45	0.9813
0.20	129.86	0.9786	126.99	0.9754	142.70	0.9786
0.25	136.62	0.9748	133.91	0.9710	150.36	0.9740
0.30	141.04	0.9773	138.45	0.9741	149.33	0.9760
0.35	142.65	0.9776	140.06	0.9743	148.30	0.9780
0.40	143.33	0.9759	140.60	0.9724	149.10	0.9775
0.45	146.10	0.9799	143.41	0.9770	151.01	0.9802
0.50	147.38	0.9800	144.73	0.9772	151.12	0.9836
0.55	147.83	0.9802	145.06	0.9772	152.14	0.9830
0.60	149.15	0.9803	146.40	0.9775	153.21	0.9832
0.65	149.59	0.9804	146.89	0.9776	154.66	0.9902
0.70	149.93	0.9787	147.14	0.9756	161.97	0.9759
0.75	151.72	0.9733	148.94	0.9694	173.92	0.9575
**Average**	140.10		137.34		148.45	
**%Variation**	22.22	**Multi-Step**	22.63	**Multi-Step**	33.05	**Multi-Step**

Table S6. Activation energy and r-squared estimated by the three isoconversional methods (FWO, KAS, FR) for Coigüe (ND) decomposition.

α	FWO		KAS		FR	
	E_a [kJ mol^{-1}]	R^2	E_a [kJ mol^{-1}]	R^2	E_a [kJ mol^{-1}]	R^2
0.05	130.38	0.9684	128.28	0.9640	125.12	0.9779
0.10	127.79	0.9768	125.31	0.9734	133.87	0.9813
0.15	129.28	0.9801	126.73	0.9771	140.51	0.9832
0.20	132.58	0.9790	130.02	0.9758	147.59	0.9804
0.25	136.55	0.9796	134.04	0.9767	153.86	0.9826
0.30	142.69	0.9791	140.35	0.9761	158.82	0.9830
0.35	149.01	0.9800	146.84	0.9771	159.76	0.9802
0.40	150.76	0.9800	148.51	0.9772	161.68	0.9844
0.45	155.03	0.9824	152.86	0.9799	161.13	0.9891
0.50	155.11	0.9855	152.90	0.9835	159.94	0.9893
0.55	156.38	0.9684	153.39	0.9856	159.73	0.9909
0.60	157.57	0.9875	155.32	0.9857	167.21	0.9911
0.65	156.05	0.9870	153.66	0.9852	169.43	0.9900
0.70	158.25	0.9867	155.88	0.9848	177.79	0.9892
0.75	162.25	0.9886	159.95	0.9870	194.81	0.9822
**Average**	144.91		144.27		158.08	
**%Variation**	23.78	**Multi-Step**	24.01	**Multi-Step**	44.09	**Multi-Step**

Table S7. Activation energy and r-squared estimated by the three isoconversional methods (FWO, KAS, FR) for Ulmo (EC) decomposition.

α	FWO		KAS		FR	
	E_a [kJ mol^{-1}]	R^2	E_a [kJ mol^{-1}]	R^2	E_a [kJ mol^{-1}]	R^2
0.05	146.24	0.9652	144.78	0.9609	122.02	0.9572
0.10	131.74	0.9845	129.25	0.9822	130.10	0.9808
0.15	130.10	0.9832	127.39	0.9806	135.16	0.9802
0.20	132.13	0.9835	129.34	0.9809	137.70	0.9782
0.25	131.83	0.9787	128.87	0.9754	138.45	0.9747
0.30	135.78	0.9794	132.84	0.9763	141.51	0.9728
0.35	136.18	0.9745	133.08	0.9706	140.36	0.9739
0.40	137.97	0.9783	134.89	0.9750	138.31	0.9833
0.45	138.57	0.9817	135.38	0.9789	135.45	0.9855
0.50	137.87	0.9831	134.50	0.9804	135.05	0.9863
0.55	138.68	0.9847	135.27	0.9822	136.20	0.9855
0.60	138.68	0.9847	135.18	0.9821	140.88	0.9858
0.65	137.56	0.9845	133.97	0.9820	144.33	0.9852
0.70	137.56	0.9845	133.87	0.9819	147.23	0.9831
0.75	137.56	0.9845	133.80	0.9818	152.88	0.9815
**Average**	136.56		133.50		138.38	
**%Variation**	11.82	**Single-Step**	13.03	**Single-Step**	22.30	**Multi-Step**

Table S8. Arrhenius reaction rate constants calculated for each biomass from A_α and E_α pairs.

α	Arrhenius reaction rate constant, k(T) [min^{-1}]				
	PR	NO	EN	EC	ND
0.05	0.0290	0.0743	0.0807	0.0534	0.0381
0.10	0.1103	0.1306	0.1550	0.1023	0.0678
0.15	0.2452	0.1975	0.2284	0.1598	0.0996
0.20	0.3963	0.2916	0.3364	0.2524	0.1380
0.25	0.5828	0.3939	0.5115	0.4017	0.1971
0.30	0.8144	0.5710	0.8498	0.6099	0.2934
0.35	1.1629	0.8334	1.2649	0.9335	0.4697
0.40	1.5955	1.2400	1.7083	1.3655	0.7207
0.45	2.0957	1.7810	2.1875	1.8761	1.0853
0.50	2.6805	2.4115	2.7162	2.4035	1.5123
0.55	3.3442	3.1237	3.2959	2.9393	1.9601
0.60	4.1053	3.8955	3.9210	3.4928	2.3643
0.65	4.9376	4.7310	4.7019	4.1398	2.9296
0.70	6.0075	5.7335	5.6704	4.9274	3.6348
0.75	7.3679	7.1355	7.2018	6.0177	4.8532

Table S9. Main pyrolytic products grouped by families [% Area] at lab-scale.

Family	PR	NO	EN	EC	ND
Alcohols	0.0	9.4	2.4	3.0	6.0
Aldehydes	1.0	1.4	0.6	2.3	0.6
Anhydro sugars	7.8	3.4	6.7	1.8	5.7
Carboxilic acids	13.0	6.9	20.7	17.1	17.1
Esters	2.2	2.1	2.0	1.1	1.2
Ethers	0.0	0.0	0.0	0.1	0.6
Furan derivatives	13.4	7.0	11.5	6.5	5.5
Hydrocarbons	0.3	3.8	0.6	1.8	3.0
Ketones	6.9	10.0	12.0	11.6	11.0
Nitro-compounds	0.8	1.4	1.3	1.5	1.7
Oxo-compounds	1.3	2.2	1.3	2.2	1.7
Phenol derivatives (lignins)	5.2	5.0	9.7	6.7	13.7
Pyran derivatives	0.8	1.3	3.3	1.1	0.9
Water	47.2	46.3	28.0	43.2	31.1

Table S10. Main compounds identified in each family.

Family	Compound
Acids	Acetic Acid<br>Propanoic acid, 2-oxo-, methyl ester
Alcohols	Acetic acid, (acetyloxy)-<br>2-Furanmethanol
Aldehydes	Furfural<br>Methyl glyoxal<br>Benzaldehyde, 4-hydroxy-3,5-dimethoxy-<br>5-Hydroxymethylfurfural<br>Acetaldehyde, hydroxy-<br>Succindialdehyde
Amines	N-Butyl-tert-butylamine<br>2-Amino-1,3-propanediol<br>Methanamine, N-hydroxy-N-methyl-<br>1-Amino-2,6-dimethylpiperidine
Ketones	2-propanone, 1-hydroxy-<br>2-Cyclopenten-1-one, 2-hydroxy-<br>2(5H)-Furanone<br>1,2-Cyclopentanedione, 3-methyl-
Esters	Propanoic acid, 2-oxo-, methyl ester<br>Methyl stearate<br>Cyclopropyl carbinol<br>1,2-Ethanediol, monoacetate<br>Acetic acid ethenyl ester
Ethers	Butane, 1,2:3,4-diepoxy-, (±)-<br>Benzofuran, 2,3-dihydro-
HC_Aromatics	3,5-Dimethoxy-4-hydroxytoluene<br>Benzene, 1,2,3-trimethoxy-5-methyl-
Phenols	Phenol, 4-ethenyl-2,6-dimethoxy-<br>2-Methoxy-4-vinylphenol<br>(E)-2,6-Dimethoxy-4-(prop-1-en-1-yl)phenol<br>Phenol, 2,6-dimethoxy-<br>Creosol<br>Phenol, 2-methoxy-4-(1-propenyl)-<br>Phenol, 2,6-dimethoxy-4-(2-propenyl)-<br>Phenol, 2-methoxy-
Sugar	Sucrose<br>β-D-Glucopyranose, 1,6-anhydro-<br>1,6-Anhydro-β-d-talopyranose<br>d-Mannose
